# Health Behaviors and eHealth Literacy Among Older Adults, HINTS 2019

**DOI:** 10.1093/geroni/igaa057.730

**Published:** 2020-12-16

**Authors:** Ruth Sanchez, Hannah Kay, Pooja Srikanth, Lyndsey Sandow, Michelle Zhang

**Affiliations:** University of Texas at Austin Dell Medical School, Austin, Texas, United States

## Abstract

With rapid shifts in how health information is reported and consumed, providers and patients must consider their electronic or “eHealth” literacy. The purpose of this study was to analyze how older adults (age 60+) seek health information in the context of online and offline resources and how eHealth literacy correlates with health behaviors. We performed a cross-sectional analysis of a nationally representative sample of 2,587 U.S. older adults drawn from the Health Information National Trends Survey (HINTS) Iteration 5 Cycle 3. Weighted descriptive analyses were conducted to examine the association between CDC-recommended health behavior guidelines on produce consumption and exercise, eHealth literacy, and sociodemographics. Weighted logistic regression analyses were conducted with STATA 16.0 to assess the relationship between healthy behaviors and eHealth literacy controlling for sociodemographics. The weighted sample reported the following demographic characteristics: average age 71 years (range 60-98), 53.6% female, 73.8% White, 9.7% Black and 8.6% Hispanic. Of older adults, 26.7% performed 2 or more health behaviors regularly. Among older adults, those who have looked up medical information using electronics are 1.79 (95% confidence interval: 1.24, 2.58) times more likely to meet 2 or more CDC-recommended health behavior guidelines as compared to those that have not, after controlling for survey group, education, race/ethnicity and gender. Access and utilization of online resources among older adults may influence their health behaviors and health outcomes. Providers should consider the eHealth literacy of their older adult patients and direct them to appropriate and reliable online resources.

